# Attaining freshwater and estuarine-water soil saturation in an ecosystem-scale coastal flooding experiment

**DOI:** 10.1007/s10661-022-10807-0

**Published:** 2023-02-24

**Authors:** A. M. Hopple, K. O. Doro, V. L. Bailey, B. Bond-Lamberty, N. McDowell, K. A. Morris, A. Myers-Pigg, S. C. Pennington, P. Regier, R. Rich, A. Sengupta, R. Smith, J. Stegen, N. D. Ward, S. C. Woodard, J. P. Megonigal

**Affiliations:** 1 Pacific Northwest National Laboratory, Richland, WA 99352 USA; 2 Smithsonian Environmental Research Center, Edgewater, MD 21037 USA; 3 University of Toledo, Toledo, OH 43606 USA; 4 Joint Global Change Research Institute, Pacific Northwest National Laboratory, College Park, MD 20740 USA; 5 Atmospheric Science and Global Change Division, Pacific Northwest National Laboratory, WA 99352 Richland, USA; 6 School of Biological Sciences, Washington State University, Pullman, WA 99164 USA; 7 Marine and Coastal Research Laboratory, Pacific Northwest National Laboratory, Sequim, WA 98382 USA; 8 California Lutheran University, Thousand Oaks, CA 91360 USA; 9 Global Aquatic Research LLC, Sodus, NY 14551 USA; 10 University of Washington, Seattle, WA 98195 USA

**Keywords:** Coastal upland forest, Ecosystem-scale manipulation, Ecosystem state transition, Estuarine water, Freshwater, Inundation, Simulated hydrologic disturbance, Soil saturation

## Abstract

Coastal upland forests are facing widespread mortality as sea-level rise accelerates and precipitation and storm regimes change. The loss of coastal forests has significant implications for the coastal carbon cycle; yet, predicting mortality likelihood is difficult due to our limited understanding of disturbance impacts on coastal forests. The manipulative, ecosystem-scale Terrestrial Ecosystem Manipulation to Probe the Effects of Storm Treatments (TEMPEST) experiment addresses the potential for freshwater and estuarine-water disturbance events to alter tree function, species composition, and ecosystem processes in a deciduous coastal forest in MD, USA. The experiment uses a large-unit (2000 m^2^), un-replicated experimental design, with three 50 m × 40 m plots serving as control, freshwater, and estuarine-water treatments. Transient saturation (5 h) of the entire soil rooting zone (0–30 cm) across a 2000 m^2^ coastal forest was attained by delivering 300 m^3^ of water through a spatially distributed irrigation network at a rate just above the soil infiltration rate. Our water delivery approach also elevated the water table (typically ~ 2 m belowground) and achieved extensive, low-level inundation (~ 8 cm standing water). A TEMPEST simulation approximated a 15-cm rainfall event and based on historic records, was of comparable intensity to a 10-year storm for the area. This characterization was supported by showing that Hurricane Ida’s (~ 5 cm rainfall) hydrologic impacts were shorter (40% lower duration) and less expansive (80% less coverage) than those generated through experimental manipulation. Future work will apply TEMPEST treatments to evaluate coastal forest resilience to changing hydrologic disturbance regimes and identify conditions that initiate ecosystem state transitions.

**Supplementary Information:**

The online version contains supplementary material available at 10.1007/s10661-022-10807-0.

## Introduction

Climate change is driving ecological shifts in coastal regions where ecosystems are particularly vulnerable to sea-level rise (Boon, 2012), salinization (Bender et al., 2010), and storm surge (St. Laurent et al., 2021). These disturbances can lead to dramatic changes in coastal forests dominated by tree species with little tolerance for low oxygen (O_2_) and/or saline conditions (Kirwan & Gedan, 2019; Spivak et al., 2019). The potential loss of coastal forests, which represent 36% of coastal land cover in the USA (Office for Coastal Management, 2022), has significant implications for the coastal carbon (C) cycle (Smart et al., 2020; Smith & Kirwan, 2021). Yet, forecasting the possibility of mortality is challenging given our limited understanding of disturbance impacts on these ecosystems (McDowell et al., 2018; Ward et al., 2020).

**Fig. 1:**
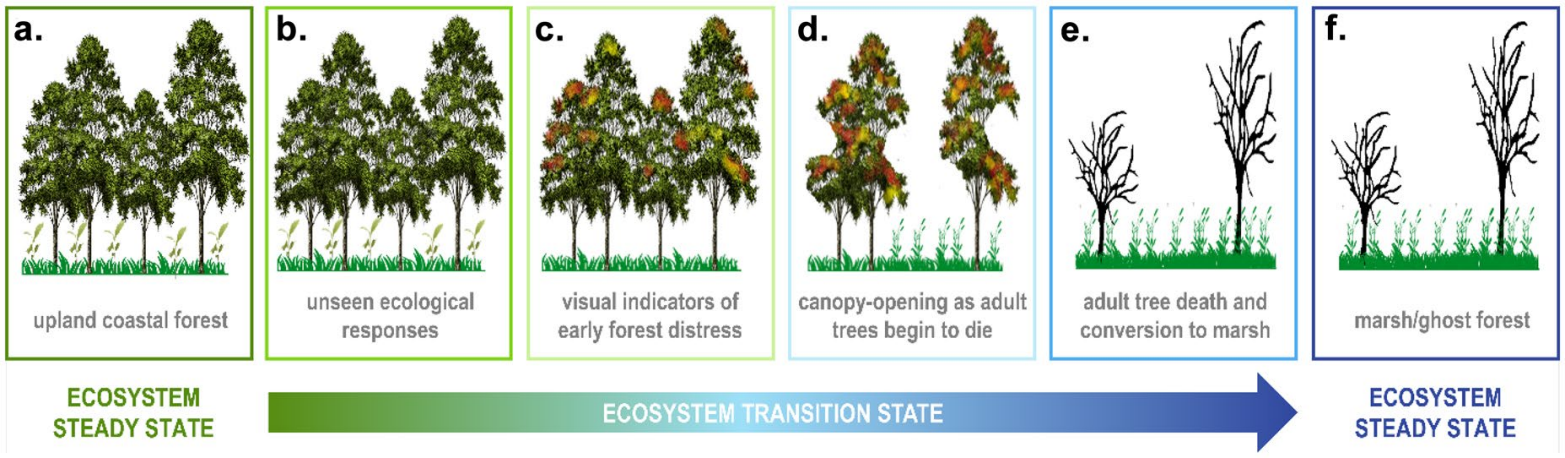
Stages of coastal upland forest transition to wetland. TEMPEST addresses the largely unknown ecological and biogeochemical changes that occur at the earliest stages of ecosystem state transitions (**b** and **c**)

The transition of coastal forests from an upland (Fig. 1a) to wetland (Fig. 1f) state often begins with storm surges that force saline water into areas with no prior exposure to salinity and/or inundation or intense precipitation that adds large volumes of freshwater to forests through direct rainfall or overbank flooding (Raabe & Stumpf, 2016; Kearney et al., 2019). The impact of such events depends on their duration, timing, frequency, antecedent conditions, and site characteristics. Subsequent transition stages may be identified by the onset of subtle ecological and/or biogeochemical responses. Lower sap flow (Teobaldelli et al., 2004) or annual growth (Begin, 1990; Fernandes et al., 2018) may be the first signs of stress in trees (Fig. 1b). More dramatic early indicators of forest distress are conspicuous young tree death and cessation of tree recruitment (Begin, 1990; Williams et al., 1999; Fig. 1c), which are followed by canopy-opening as mature trees die (Williams et al., 1999) and salt-tolerant species establish themselves (often *Phragmites* and shrubs; Conner et al., 2007; Langston et al., 2017; Fig. 1d); and finally, tree death and conversion to marsh (Li et al., 2021; Fig. 1e). Ecological dynamics and biogeochemical cycling in these distinct ecosystems—upland forests and tidal wetlands—are well characterized at steady state (Fig. 1a, f), but little is known about the intricate series of events that occurs as forests transition to a tidal wetland state (McDowell et al., 2018; Ward et al., 2020).

Hydrologic disturbances that increase soil saturation, whether freshwater or saline, interrupt the soil-plant-atmosphere dynamics of upland forests. Saturation does not directly affect canopy gas exchange, but it severely restricts exchange of O_2_ between the atmosphere, soil pore spaces, and tree roots. Molecular O_2_ is consumed rapidly by aerobic plant and microbial respiration in soils and tree stems and can only be replaced by atmospheric gas flux through the connected continuum of pore spaces that connect soils and tree stems (Swift et al., 1979; Moyano et al., 2013). As these spaces fill with water, diffusion pathways are effectively blocked as O_2_ diffusion rates decline 10^3^-fold and [O_2_] declines (Skopp et al., 1990). Subsequent development of hypoxic or anoxic conditions fundamentally alters the dominant pathways of microbial respiration, both in soils (Megonigal et al., 1993, 2004) and in tree stems (Covey & Megonigal, 2019), and negatively impacts the physiology of plants. Filling pore spaces with saline water amplifies the physiological stresses of inundation by decreasing water uptake in woody plants (Boursiac et al., 2005) and increasing osmotic stress on plants and microbes (Sutka et al., 2011; Stavridou et al., 2017). As the frequency of transient floods increases, plant and microbial impacts accumulate until one or more critical thresholds are crossed and the forest declines without recovery (Hammond, 2020).

Our poor understanding of coastal forest responses arises from a striking paucity of data and from the challenge of accurately capturing event-focused (e.g., storm surge, drought, fire) ecological and biogeochemical disturbance impacts at ecosystem scales. This type of research is difficult because extreme events that induce abrupt ecosystem state transitions are typically spatially and temporally unpredictable, and it is not logistically feasible and/or safe to sample during such events. Thus, post-event observational studies often lack relevant pre-treatment data and/or are unable to discern mechanistic drivers due to correlated and interacting perturbation effects (Rogers et al., 2012; Fayle et al., 2015). Additionally, the lack of reliable recurrence intervals severely limits the estimation of key ecosystem tipping points. While long-term coastal research networks are poised to observe extreme events, these sites generally focus on surface water and marsh dynamics as opposed to coastal forest transitions (Hopkinson et al., 2008).

Given these constraints, large-scale experimental manipulations have been proposed as an effective approach for assessing event-focused disturbance impacts on ecosystems as they allow control over disturbance frequency, intensity, and timing (Jentsch et al., 2007; Hanson & Walker, 2020). Manipulative, ecosystem-scale field experiments have an extensive history of enabling researchers to discern complex mechanistic drivers and expediting the development of predictive models in ecosystems experiencing trend-based environmental change (e.g., warming, elevated atmospheric CO_2_; Hanson & Walker, 2020). Applying such experimental frameworks to advance event-based research is a relatively recent trend (Jentsch et al., 2007) and we are unaware of any comparable efforts that address hydrologic disturbance impacts on coastal forests.

Here, we describe the rationale for the design, implementation, and performance of the Terrestrial Ecosystem Manipulation to Probe the Effects of Storm Treatments (TEMPEST) experiment that we developed to simulate hydrologic disturbance events in a coastal upland forest. This experiment addresses the potential for freshwater and estuarine-water disturbance events to alter tree physiology, species composition, and ecosystem processes in a deciduous coastal forest in MD, USA. Forests are among the most challenging places to conduct manipulative experiments because interactive responses to environmental perturbation occur at large spatial scales and herbaceous and woody plant structural variation creates spatially heterogeneous above and below-ground conditions, complicating the control and uniformity of experimental treatments. Therefore, TEMPEST was designed to generate hydrologic disturbance events across 2000 m^2^ coastal forest plots (i.e., large spatial scale) using a spatially focused water application approach (i.e., uniform treatment application).

## Methods

### Site description

Our study site is located on the western shore of the Chesapeake Bay in MD, USA at the Smithsonian Environmental Research Center (SERC) and adjacent to the Global Change Research Wetland (GCReW: https://serc.si.edu/gcrew). Over the period from 1986 to 2019 the average annual air temperature was 11.9 °C, with monthly mean extremes of −6.0 and 33.6 °C, and the average annual precipitation was 949 mm, with maximum daily precipitation as high as 229 mm (meteorological data sourced from Annapolis Naval Academy weather station; 13.5 km NE of TEMPEST). The 226-ha forested watershed is drained by a second-order stream that flows into a brackish tidal marsh with a salinity range of 4 to15 psu (mean = 10 psu) and a mean tidal range of 44 cm. Our 2000 m^2^ experimental plots are 5 m higher and ~ 25–50 m away from the shoreline and have no known prior exposure to seawater. For instance, they were not inundated during the historically large storm surge from Hurricane Isabel in 2003 (J. P. Megonigal, per. comm.).

The TEMPEST experiment is in a mid- to late-successional (~ 80 years old) temperate, deciduous coastal forest. Deciduous forest covers 12.6% of the coastal USA and 23.5% of the coastal mid-Atlantic region (Office for Coastal Management, 2022). The closed canopy is dominated by *Liriodendron tulipifera*, *Fagus grandifolia*, *Acer rubrum*, and *Quercus* spp. All saplings greater than 1 cm diameter at 1.3 m (diameter at breast height, DBH) above the soil surface are defined as trees for this study. The mean plot tree diameter is 22.9 ± 1.7 cm and the mean number of total and larger (DBH ≥ 20 cm) trees per plot are 138 ± 16 total trees and 60 ± 12 large trees, respectively (Supplemental Table 1). There is very little structure to the understory, but it contains small stature *Ilex opaca* and several deciduous shrubs, including *Rubus phoenicolasius*, *Lindera benzoin*, *Berberis thunbergii*, and *Elaeagnus umbrellata*. The forest also supports an herbaceous layer mainly composed of *Mitchella repens*, *Polygonum virginianum*, *Rhus radicans*, *Symphyotrichum lateriflorus*, *Epifagus virginiana*, and *Galium circaezans*, as well as a small number of woody vines such as *Lonicera japonica* and *Parthenocissus quinquefolia*.

**Table 1:** Comparison of average soil volumetric water content (VWC) response at 5, 15, and 30 cm below ground following experimental and natural hydrologic disturbance events

		Peak soil VWC		Time to peak soil VWC (h)		Duration of peak soil VWC (h)		Time to return to ambient baseline (h)	
Plot	Depth (cm)	Simulated	Natural	Simulated	Natural	Simulated	Natural	Simulated	Natural
FW	5	0.42	0.40	7	5	5	1	~ 10	~ 10
	15	0.39	0.37	6	5	7	3	~ 10	~ 10
	30	0.41	0.41	4	5	20	3	~ 24	~ 24
EW	5	0.43	0.37*	7	3	5	1	~ 10	~ 4
	15	0.37	0.34*	7	4	6	2	~ 10	~ 5
	30	0.37	0.35	5	7	14	2	~ 24	~ 10

FW = freshwater plot; EW = estuarine-water plot; Simulated = TEMPEST experimental manipulation; Natural = Hurricane Ida; * = was not saturated

The soils are classified as Typic Hapludults and based on ground-penetrating radar and soil core characterization, consist of 3 distinct sediment horizons that are separated by gradual changes in soil coarseness (i.e., particle size; Supplemental Fig. 1). Rooting zone soils (0 to 30 cm) are well-drained, fine sandy loams that are high in organic matter (OM) content and are underlain by a silty clay layer (to a depth of 1.6 m). Clay content increases with depth throughout the upper sediment horizons and is greatest at 1.6 m. Soil texture transitions from silty clay to silty sand at ~ 1.6 m, separating the second and third sediment horizons. Sand content increases throughout the third horizon and to a depth of 3.5 m.

### Experimental design

**Fig. 2:**
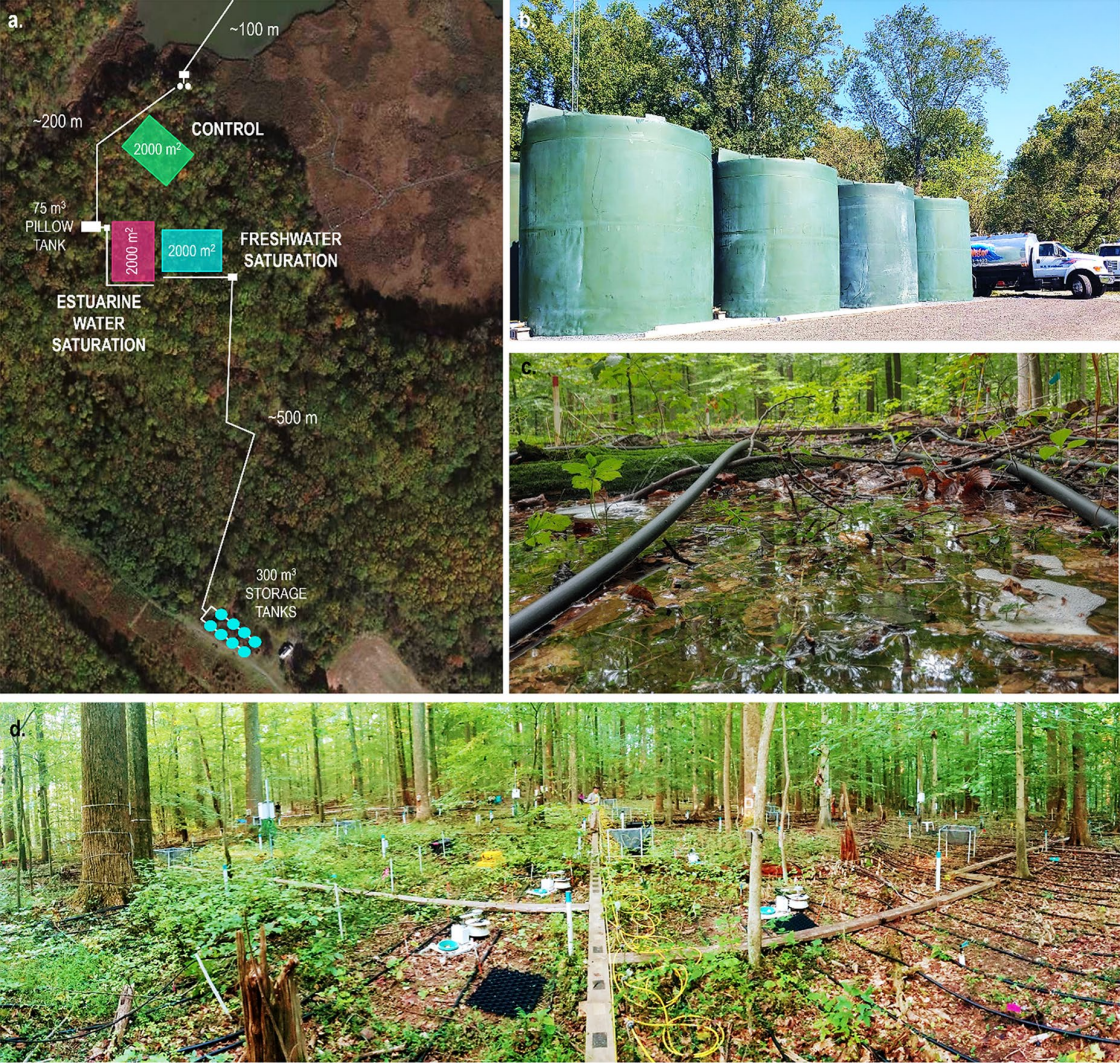
Aerial view of the TEMPEST site (**a**), photo illustrating the volume of water (only half of the storage tanks are shown) added during a single TEMPEST event (**b**), photo showing the low-level inundation observed during simulation events (**c**), photo capturing forest structure, experimental infrastructure, and grid-style spatial layout (**d**)

The TEMPEST experiment simulates extreme, ecosystem-scale freshwater and estuarine-water disturbance events using a novel, large-unit (2000 m^2^), un-replicated experimental design, with three 50 m × 40 m plots serving as control, freshwater, and estuarine-water treatments (Fig. 2a). A high-resolution spatiotemporal approach is used to monitor the impacts of experimental treatments on hydrologic drivers (e.g., soil moisture, groundwater level) and biological response variables (e.g., sap flow, soil respiration) to (1) detect ecosystem state changes (e.g., Fig. 1b–e) and (2) discern response mechanisms. Spatial dynamics are largely addressed with a grid-system strategy that quantifies within plot spatial variability and coordinates measurements spanning the soil-plant-atmosphere continuum (Fig. 2d). Temporal patterns are captured by continuous sensor networks and maximizing discrete measurement collection frequencies, particularly immediately prior to, during, and following simulation events.

Simulated hydrologic disturbance events will begin at a low frequency to avoid severe stress on the trees that would lead to rapid mortality. Decisions regarding the frequency of additional events will be based on the measured and modeled tree physiological responses to the first event and each event thereafter. Ultimately, we will increase event frequency as needed to challenge the physiological capacity of trees to flooding and salinity, with the goal of inducing significant tree mortality over a decade.

Biological response data from TEMPEST will be analyzed using a before-after-control-impact (BACI) approach. BACI designs are widely considered to be the optimal method for detecting ecological perturbation effects because they incorporate both before-impact and control site data, reducing the likelihood that unknown covariates are driving observed responses (Smokorowski & Randall, 2017). Ecologists routinely use this approach when studying the ecological and biogeochemical impacts of perturbation and restoration in aquatic (de Mutsert & Cowan, 2012; Larson et al., 2018, 2019) and terrestrial (Christianson & Creel, 2014; Klaus et al., 2018) ecosystems for which replication is difficult or impossible. The successful implementation of this approach hinges upon adequate pre-treatment data from the impacted area and an un-disturbed control area. Pre-treatment data collection began in 2019 and the first paired freshwater and estuarine-water simulation events are scheduled for June 2022. This paper focuses on simulation events (described below) that were conducted in September 2021 to assess the performance of TEMPEST infrastructure in preparation for the official experiment launch.

### Water delivery procedures

Our water delivery infrastructure was designed, built, and tested by Global Aquatic Research LLC (https://www.globalaquaticresearch.com/index.html). The freshwater and estuarine-water treatment plots have identical water application systems but differ in their sourcing, storage, and processing of water. The freshwater (FW) system receives and stores 300 m^3^ (300,000 l) of municipal water in eight 40 m^3^ polyethylene tanks (Snyder Industries) that are connected to a manifold which accommodates tank filling and discharge. The FW is fed through a ~ 500 m pipeline to a gas-powered pump (~ 8 cm high-pressure pump, Empire Drip Supply) at the corner of the FW plot. The manifold and pipeline are constructed of ~ 8 cm and 10 cm PVC connected by glued PVC couplings at 6 m intervals. Air vents and threaded PVC unions were installed at certain locations to prevent airlocks within the line and to facilitate drainage and cleanout. Downstream of the pump, the FW passes through a disc filter, a water meter, and a 50 PSI pressure regulator before distribution throughout the FW plot via a network of irrigation tubing equipped with pressure-compensating emitters. The FW system is also able to connect to the estuarine-water (EW) system to rinse after each simulation to prevent salt crystal accumulation following treatment applications.

The EW system sources the corresponding 300 m^3^ of estuarine water from the nearby Rhode River estuary, which has an average salinity of ~ 10 psu. A stainless-steel cylindrical intake with 1 mm slot openings (30 cm high × 79 cm diameter) is deployed from a modified barge and water is drawn from a depth of 70–80 cm at high tide. The intake system shuts down when the water depth is ≤ 40 cm; however, the maximum pumping rate from the estuary is ~ 70% more than the rate of water supplied by the EW irrigation system and combined with a 75 m^3^ storage system (described below), the EW system can continuously supply water to the irrigation network despite being shut off during the lowest tides.

EW is drawn through the intake into a 6 m section of 10 cm PVC pipe strapped to the underside of the barge. It is drawn through ~ 100 m of suspended 10 cm PVC pipe to the shoreline by a gas-powered pump (~ 8 cm high-pressure pump, Empire Drip Supply). The EW is then passed through an agricultural filtration system (Yardney, two 76 cm SM350 filters) to remove particles > 1 mm in diameter and transported uphill through a ~ 200 m pipeline into a 75 m^3^ pillow tank (custom designed by GAR, manufactured by Husky Portable Containment). An ~ 8 cm high-pressure pump draws water from the pillow tank to the corner of the EW plot, where it is passed through a secondary disc filter, water meter, and pressure regulator, prior to distribution through an irrigation system identical to that of the FW plot.

The irrigation network of both systems consists of a ~ 8 cm header line that spans the 50 m length of each treatment plot and connects to 40 m long, ¾” irrigation lines spaced 0.5 m apart (*n* = 100 irrigation lines per treatment plot). Each irrigation line contains 40 pressure-compensating emitters spaced 1 m apart, with placement offset by 0.5 m between adjacent lines to reduce the distance between emitters. Emitters release 8 l of water per hour. The water delivery rate is just above the drainage capacity of the soil (based on infiltration tests and tests from scaled-down iterations of the irrigation system, data not shown) and was selected to maximize the time that the soil remains saturated while minimizing water loss by surface runoff.

The time required to achieve soil saturation and the duration of saturation will vary temporally based on seasonal changes in soil moisture conditions as would be the case for naturally occurring extreme saturation events. If necessary, we can increase the water delivery capacity of our system by extending the treatment application time. This is accomplished by coordinating mid-treatment deliveries of freshwater to refill the eight 40 m^3^ FW storage tanks and replenishing the 75 m^3^ EW pillow storage tank more than four times. This system flexibility increases our likelihood of attaining saturation even under extremely dry conditions and high soil infiltration rates.

### Environmental monitoring for performance evaluation

We tested the effectiveness of our water delivery system infrastructure by completing two freshwater saturation events, one each in the freshwater and estuarine-water treatment plots on August 25 and September 9, 2021, respectively. Our goals were to assess our operational ability to execute simulated hydrologic disturbance events and to determine the spatial and temporal extent of treatment impacts on hydrologic drivers. *Only freshwater* was used for this evaluation because a single application of freshwater was unlikely to produce lasting effects in this system, while novel exposure to estuarine water may have generated lasting biotic and/or abiotic impacts, confounding our experimental design. For both events, we aimed to deliver 300 m^3^ of freshwater to each treatment plot at an average rate of 640 l per minute (LPM) over a 10-h period. Coincidentally, the remnants of Hurricane Ida hit the TEMPEST site in the week between these two events (i.e., September 1, 2021), allowing us to compare simulated and natural hydrologic disturbance impacts under similar ambient conditions.

TEMPEST treatment impacts on hydrologic drivers were assessed using a combination of soil volumetric water content (VWC), groundwater level, and subsurface electrical resistivity measurements. A spatially distributed sensor network (TEROS 12, Meter) was used to measure soil VWC (manufacturer-reported range = 0.0–0.7 m^3^/m^3^; accuracy =  ± 3%^)^ at 15-min intervals and at multiple depths (5, 15, and 30 cm below ground) in each plot to capture high resolution spatial and temporal treatment patterns. The same sensors also measure electrical conductivity (which will be used to track eventual estuarine-water applications; range = 0–20,000 µS/cm; accuracy =  ± 5–8%) and temperature (range =  −40–60 °C; accuracy =  ± 0.5%). In each plot, soil sensors were installed at 15 cm in 65% of the interior 25 m^2^ grid cells (*n* = 31 measurement locations per plot) and at 5 and 30 cm in 5 of these locations (*n* = 5 depth profiles per plot).

Groundwater level was measured using Aqua Troll 600 multiparameter sondes (In Situ, UK) deployed in groundwater wells at the center of each experimental plot to capture hydrologic linkages between surface flooding and groundwater table dynamics.

Electrical resistivity measurements along parallel 2D transects served as proxy for characterizing the shallow subsurface architecture (including soil types and spatial extent) and for monitoring changes in water saturation during the freshwater plot simulation event (data for estuarine water plot are not available). This was done by injecting direct current into the subsurface using a pair of electrodes and measuring the resulting potential difference with another pair of electrodes. Based on Ohm’s law and considering the electrode arrangement, an average or “apparent” electrical resistivity was calculated (Singha & Gorelick, 2005; Binley & Slater, 2020). Using the apparent resistivity distribution, the true resistivity distribution of the subsurface is estimated by solving the governing equation (i.e., the Poisson’s equation for electrical potential distribution via an iterative inversion; Binley & Slater, 2020). Field resistivity data were acquired with an R8 SuperSting resistivity meter (AGI USA, Austin, TX) and a multi-electrode switchbox which allows an automatic switching of up to 84 current and potential electrodes. The data were acquired along 3 parallel transects at both edges and the middle of the freshwater plot prior to treatment. Continuous monitoring along the middle transect during the treatment was used to monitor infiltration fronts. Each transect included 84 stainless steel surface electrodes using a dipole–dipole electrode configuration and a 0.5 m unit electrode spacing resulting in a profile length of 41.5 m. Current injection was set at 1.2 ms with 3 measurement cycles of the resulting potential difference and measurement error set at 2%. Over 52 min, 1453 apparent resistivity data points were acquired along each transect. The same resistivity meter was used for both transects resulting in a total measurement time of 110 min per time-lapse cycle.

### Data processing and analysis for performance evaluation

#### Soil VWC

Daily average soil temperature and VWC for each sensor were calculated using 15-min interval data (sensor number = 137; measurements per sensor per day = 96) to characterize the ambient conditions preceding hydrologic disturbance events. We visualized interannual seasonal trends in these variables using time-series graphs that spanned May 2020 to December 2021. Trends were separated by depth but included data from all experimental plots because treatment applications had not yet started.

We determined the spatial extent of hydrologic disturbance impacts (simulated and natural) on soil VWC by assessing individual soil sensor responses (lateral variation) at each depth increment (vertical variation). Mean changes in soil VWC at each depth were calculated at hourly intervals for the 24-h period following the start of water delivery to quantify the temporal dynamics of hydrologic disturbance impacts (simulated and natural) on soil VWC. Saturation was indicated by soil VWC measurements that plateaued near 0.4 m^3^/m^3^ (based on soil infiltration tests, data not shown). Failure to achieve soil saturation could result if the water delivery magnitude and rate constraints inherent in our system design do not surpass infiltration rates, which can vary with antecedent precipitation and evapotranspiration rates. Values outside of the manufacturer-issued range were removed for all analyses.

#### Groundwater level

To calculate groundwater level, pressure (range = 0.0–9.0 m, accuracy = 1% of full scale) measured by each sonde was corrected for atmospheric pressure measured at a nearby meteorological station, water density measured by the sonde, and the distance between the ground surface and the pressure sensor of each sonde.

#### Electrical resistivity

Measured apparent resistivities were first filtered to exclude data with errors exceeding the 2% error range for stacked 3 different potential measurement cycles for each current injection. This resulted in excluding an average of 4% of the data in each measured 2D transect. Thereafter, the resistivity data acquired prior to the treatment application were inverted using a finite element-based Gauss–Newton numerical scheme implemented in ResIPy (Blanchy et al., 2020) to generate 2D resistivity distributions for each of the 3 transects. The inversion minimizes the misfit between measured and theoretical resistivity distribution while enforcing a smoothness constraint (Binley & Kemna, 2005). A time-lapse resistivity inversion using a difference inversion approach was also implemented in ResIpy to highlight the difference in resistivity between each successive time step and measurement prior to treatment taken as background resistivity data. Similar to the standard inversion, the time-lapse inversion minimizes the misfit between the difference between two datasets and the difference between two model responses.

## Results

### Execution of hydrologic disturbance simulation

We successfully delivered 307 and 291 m^3^ of freshwater at an average rate of 640 LPM to the TEMPEST freshwater (August 25, 2021) and estuarine-water (September 9, 2021) experimental plots, respectively (Fig. 2b provides a photo that may help the reader visualize this volume of water). Both treatment applications were completed over the course of 10 h, with water addition beginning at 7:00 AM and ending at 5:00 PM. Water delivery proceeded as anticipated and culminated in widespread, low-level inundation (~ 8 cm of standing water) of the freshwater and estuarine-water plots (Fig. 2c).

### Ambient conditions preceding simulated hydrologic disturbance

Our simulation events took place immediately after peak annual temperatures began to decline (Supplemental Fig. 2a) and as soil moisture content began to increase from its annual minimum (soil VWC as low as 0.12) following a seasonal water table drawdown in early August 2021 (Supplemental Figs. 2b and 3). Although our two simulation events were conducted 22 days apart, the ambient environmental characteristics were nearly identical and were consistent with those measured prior to the arrival of Hurricane Ida (Supplemental Table 2). Mean daily soil temperature and VWC at 15 cm ranged from 21.5 ± 0.03 to 23.8 ± 0.03 °C and 0.33 ± 0.01 to 0.34 ± 0.01 m^3^/m^3^ and groundwater level varied by less than 1% across the 3 days preceding hydrologic disturbance events.

**Fig. 3:**
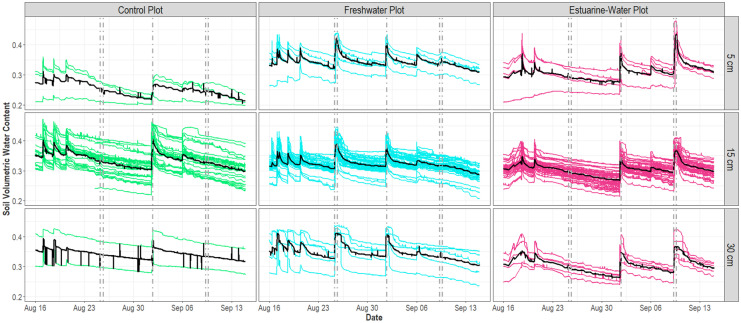
Time-series of soil volumetric water content at 5, 15, and 30 cm below ground in the control (left column), freshwater (center column), and estuarine-water (right column) plots during experimental and natural hydrologic disturbance events. Individual and average responses from the spatially distributed soil sensor network (5 cm: *n* = 5, 15 cm: *n* = 31, 30 cm: *n* = 5) are shown as colored and black solid lines, respectively. TEMPEST simulations are indicated by the paired dotted lines (i.e., first pair = freshwater flood in freshwater plot; second pair = freshwater flood in estuarine water plot) and Hurricane Ida is designated by the single dotted line

Two-dimensional resistivity models supported prior soil profile characterization (i.e., Supplemental Fig. 1, described in the “Site description” section) by showing that subsurface architecture (0–5 m below ground) was composed of 3 distinct soil layers (Supplemental Fig. 4; data only available for freshwater plot). Under ambient conditions in the freshwater plot, resistivity values were highest in sandy loams (first layer, 0–0.5 m) and lowest in silty clays (second layer, 0.5–1.6 m), with silty sands (third layer, 1.6–3.5 m) exhibiting resistivities that were in between these extremes. This subsurface architecture was continuous throughout the middle section of the freshwater plot but disrupted near the southern and northern plot boundaries where sandy loams (layer 1) were thicker and silty clays were minimal (layer 2).

**Fig. 4:**
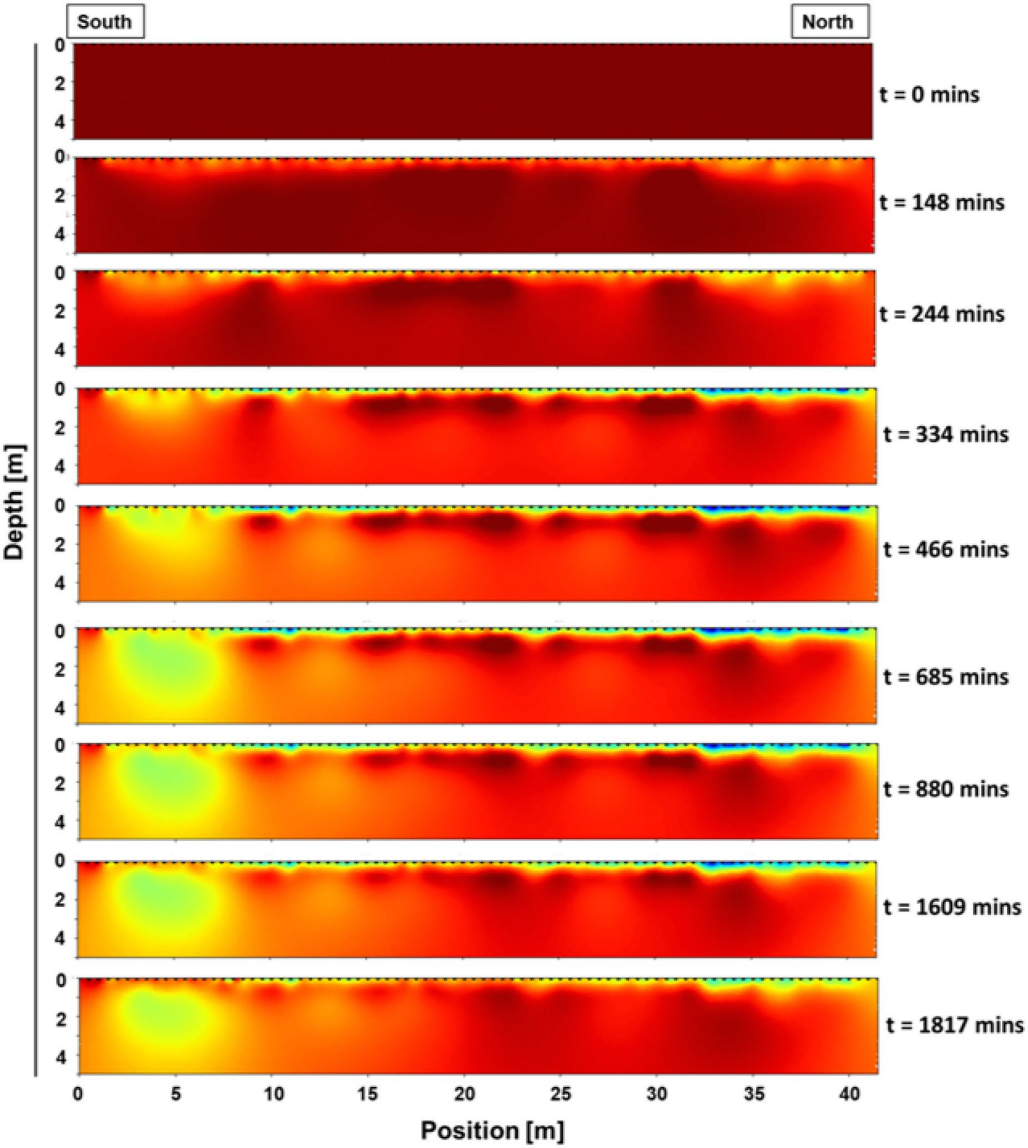
Selected time-lapse images showing resistivity changes to a depth of 5 m below ground in the freshwater plot during a TEMPEST hydrologic disturbance event. Measurements were made along a 40 m transect that was established in the center of the experimental plot. Ambient soil water content is shown in dark red (top panel), soil moisture increases are indicated by lighter red to yellow colors (panels 2 to 9), and soil saturation is designated by blue colors (restricted to upper 50 cm of soil profile, panels 4 to 8)

### Spatial and temporal extent of simulated hydrologic disturbance

Soil VWC increased and plateaued for the entire spatially distributed sensor network (0–30 cm) during the 10-h freshwater application in both the freshwater and estuarine-water plots (Fig. 3). Similarly, subsurface resistivity monitoring showed wetter conditions in the upper 50 cm soil profile along the entire 40-m length of the freshwater plot after 6–8 h (Fig. 4, *t* = 334 to 466 min). We observed no corresponding changes in the control plot soil VWC during either TEMPEST simulation event (Fig. 3).

Initial increases in soil VWC were simultaneous across depth increments and saturation was attained within ~ 7 h at 5 and 15 cm and within ~ 4.5 h at 30 cm (Supplemental Table 3). Saturation of soils at 5 and 15 cm lasted for 5 and 6.5 h on average, with moisture conditions at both depths gradually returning to an ambient baseline over the course of 10 h. The greatest treatment impacts on soil VWC were observed at a depth of 30 cm which remained saturated for 14 to 20 h and then slowly declined and stabilized over the following day. We found analogous patterns in soil resistivity where peak responses (up to 35% decrease) occurred in the predominant rooting zone (< 50 cm) and persisted for several hours but no more than 1 day (Fig. 4, *t* = 1817 min).

**Fig. 5:**
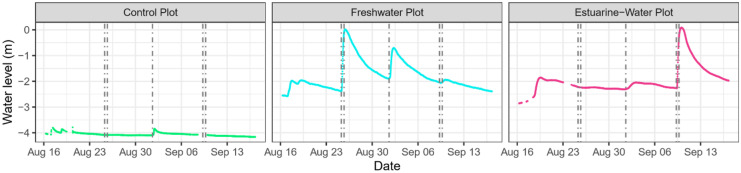
Time-series of groundwater level in the control (left panel), freshwater (center panel), and estuarine-water (right panel) plots during experimental and natural hydrologic disturbance events. TEMPEST simulation events are indicated by the paired dotted lines (i.e., first pair = freshwater flood in freshwater plot; second pair = freshwater flood in estuarine water plot) and Hurricane Ida is designated by the single dotted line

Hydrologic responses at depths greater than 0.5 m below ground, were detected within 1 to 3 h of experimental water addition and remained for several hours after delivery had ended (Fig. 4, *t* = 244 to 1817 min; Fig. 5). During this period, groundwater levels rose from a depth of ~ 2.3 m below ground to the surface in both the freshwater and estuarine-water plots (Fig. 5). Resistivity changes were largely restricted to the upper 2 m of the soil profile; however, 18% decreases in resistivity were documented 5 m below ground in the southern section of the freshwater plot (Fig. 4, *t* = 685 to 1817 min). Control plot groundwater level did not fluctuate during either experimental disturbance event (Fig. 5).

### Comparison of experimental and natural hydrologic disturbances

Hurricane Ida resulted in hydrologic impacts that were of lesser spatial extent and shorter duration than those of simulated events and unlike simulated events, also impacted hydrologic drivers in the control plot (Figs. 3 and 5). Notably, Hurricane Ida did not saturate soils at 5 and 15 cm in the estuarine-water plot and, when saturation did occur, its persistence was 2–7 times shorter than that of TEMPEST simulations (Table 1). Similarly, Hurricane Ida increased groundwater levels across all plots, but the impact magnitude (i.e., change in groundwater position) was substantially lower than that of simulated hydrologic disturbance events (Fig. 5). Furthermore, we note that Hurricane Ida generated distinct groundwater responses between the freshwater and estuarine-water plots (i.e., a large, clear peak for in the freshwater plot but a shallower, muted peak in the estuarine-water plot), consistent with soil VWC measurements indicating higher spatial variability during natural disturbance events.

## Discussion

Overall, the TEMPEST water delivery infrastructure was able to successfully apply 300 m^3^ of freshwater across each 2000 m^2^ experimental plot, with an even spatial distribution over 10 h. Our results thus show that the system can effectively attain soil saturation to a depth of at least 30 cm in an upland coastal forest by delivering the water at a rate just above the infiltration rate of the soil. Here, we address the ecological context and implications of our results.

### Magnitude of TEMPEST hydrologic disturbance

The application of 300 m^3^ of water over a 2000 m^2^ area approximates a 15 cm rainfall event. The average monthly precipitation for the area ranges from 2.1 to 7.5 cm and the average annual precipitation is 94.9 cm (meteorological data sourced from Annapolis Naval Academy weather station; 13.5 km NE of TEMPEST). Thus, a single TEMPEST simulation delivers roughly 40 times more water than the daily average precipitation during the wettest month and represents 16% of the average yearly total precipitation.

Over the last 33 years, the forested watershed surrounding the TEMPEST experiment has experienced only 4 rainfall events that delivered ≥ 15 cm of precipitation within 1 day (range = 14.9–22.9 cm; occurred in 1996, 1999, 2010, and 2012). Three of these cases were caused by intense precipitation from tropical storms. For example, Hurricane Floyd was a category 4 major hurricane that struck the Delmarva Peninsula as a tropical storm on September 15, 1999. The maximum rainfall recorded in Maryland was 35 cm (rainfall for TEMPEST area = 21.1 cm) and subsequent extreme river flooding caused $7.9 million of damage throughout the state (Tallman & Fisher, 1999). Similar massive amounts of rainfall accompanied Tropical Storm Nicole’s arrival in Maryland on September 30, 2010 (rainfall for TEMPEST area = 22.4 cm). Finally, Hurricane Sandy, the largest Atlantic hurricane on record, made landfall on October 29, 2012, and record-breaking rainfall was experienced across Maryland (rainfall for TEMPEST area = 14.9 cm). The hurricane’s storm surge affected coastal ecosystems throughout the Chesapeake Bay, particularly along the eastern shore where wind-driven surges of ~ 1-m pushed saline waters up into the headwaters of rivers and small bays, and fringing brackish marshes (Yeates et al., 2020). Thus, the hydrologic disturbance intensity of one TEMPEST event is comparable to that of a 10-year storm for the area.

### Spatial and temporal extent of TEMPEST hydrologic disturbance

The TEMPEST experiment achieved transient saturation (at least 5 h) of the entire soil rooting zone (0–30 cm) across each 2000 m^2^ forested treatment plot. Temporal patterns in soil VWC were spatially consistent and restricted to experimentally manipulated plots (i.e., no response in the control plot). The time required to reach saturation decreased with depth, while the duration of saturation increased with depth. Saturated soils at 5 and 15 cm returned to baseline conditions in roughly half the time it took soils at 30 cm. Depth-specific water relationships such as these are likely driven by silt and clay content which reduces infiltration rates and lengthens water residence time in soils (Yesilonis et al., 2016). Indeed, the silt and clay content of TEMPEST soils increased with depth throughout the major rooting zone and culminated in a distinct clay-enriched layer ~ 50 cm below ground, supporting our assertion that such features likely regulate soil VWC spatial and temporal dynamics following simulated hydrologic disturbance events.

While experimental disturbance events had the greatest impact on hydrology at 0–50 cm, groundwater monitoring and resistivity measurements verified that treatment effects extended as deep as 5 m below ground. Resistivity models indicated that preferential infiltration pathways occurred where the 50-cm clay-enriched layer was discontinuous, suggesting that treatment water may have first impacted groundwater tables near these areas (e.g., the southern edge of the freshwater plot). This heterogeneous soil structure varied the timing and extent of early hydrologic responses; however, independent, spatially distributed measurements of groundwater level were quite uniform after approximately 3 h of water delivery. This was supported by the synchronous timing of widespread inundation at the surface and the maximum elevation of the groundwater table.

### TEMPEST and Hurricane Ida

Hurricane Ida struck Louisiana as a category 4 major hurricane with sustained winds of 150 mph on August 29, 2021. On September 1, 2021, the storm remnants arrived at our study site and gave us the opportunity to compare the spatiotemporal dynamics of experimental and natural disturbance events. Hurricane Ida’s effects on hydrologic drivers were comparatively brief (80% shorter) and small (40% less spatial coverage) relative to those produced by TEMPEST simulations. However, disturbance mode (i.e., experimental vs natural) was not the primary factor underlying these differential effects, rather they were mainly caused by substantial differences in the magnitude of water added during each disturbance event. The remnants of Hurricane Ida delivered approximately 3-times less water than a TEMPEST simulation (Hurricane Ida = 5.1 cm; TEMPEST = 15 cm), with a total rainfall amount that was in-line with the historic average of maximum daily precipitation (3.4 cm ± 2.7 cm). Hurricane Ida’s spatiotemporal impacts on soil hydrology were likely further reduced by factors such as canopy rainfall interception and inherent spatial stochasticity. Overall, the hydrologic disturbance intensity of TEMPEST was much greater than that of Hurricane Ida, supporting the characterization of experimental treatments as equivalent to a 10-year storm for the area.

## Conclusion

Our results demonstrate that the TEMPEST experiment will enable us to simulate and control the frequency and quality (freshwater or estuarine water) of extreme, ecosystem-scale hydrologic disturbance events in a coastal upland forest. Future work will apply TEMPEST treatments to evaluate coastal forest resilience to changing hydrologic disturbance regimes and identify conditions that initiate ecosystem state transitions. We hypothesize that the effects of short-term soil saturation events with freshwater will be small but increase as the frequency of such events increases, while the effects of soil saturation with estuarine water will accumulate with successive exposures and will accrue with repeated short-term applications. These changes will be initiated by shifts in the fraction of ecosystem pore space that is either gas- or water-filled, which in turn will alter the redox status of both soils and trees, favoring anaerobic soil biogeochemistry and creating plant stress. Significant environmental variable shifts will coincide with the current stage of ecosystem state transition. We expect that gradual increases in water-filled ecosystem pore space and salinity will produce, with some lag, progressive decreases in transpiration and net primary productivity as successive soil saturation events occur. Eventually, we anticipate that chronic stress from compounding extreme hydrologic disturbance will exceed the impact threshold of coastal upland forests, resulting in tree mortality and an ecosystem state transition.

The TEMPEST experiment is poised to test the above hypotheses at an ecologically relevant scale and without site-to-site confounding factors to provide crucial mechanistic linkages between purely observational studies, data synthesis efforts, and smaller-scale field and laboratory manipulations. Modeling efforts are also critical to the TEMPEST experiment to both inform treatment applications and to facilitate further model development by providing the empirical data necessary for model parameterization and evaluation. TEMPEST will provide empirical data on the short- and long-term impacts of freshwater and estuarine-water disturbance events on environmental drivers (e.g., water potential, electrical conductivity, water chemistry), biological responses (e.g., sap flow, tree growth, stand density), and response mechanisms (e.g., linear tracking, threshold, hysteresis) in coastal forests. This mechanistic data can be used to parameterize demographic process models that include detailed plant physiological processes, allowing for mechanistic representation of plant recruitment, growth, and survival. This tight coupling of models, model-derived hypotheses, observations, and experimentation will enhance our predictive understanding of coastal systems and their responses to short- and long-term environmental change.

## Supplementary Information

Below is the link to the electronic supplementary material.

Supplementary file1 (DOCX 3333 KB)

## Data Availability

All code and data necessary to reproduce our results are available in our online GitHub repository (https://github.com/COMPASS-DOE/TEMPEST) and permanently archived at Pacific Northwest National Laboratory’s DataHub. Meteorological data were compiled from high-quality meteorological stations near the Global Change Research Wetland. The closest station is 13.5 km from the GCREW site at the Naval Academy in Annapolis, MD. When there were gaps in this dataset, we used data from a station named Police Bar, which was also located in Annapolis. Finally, gaps in the Annapolis datasets were filled with data from the Baltimore Washington Airport station, located 36 km from the GCREW site. All data were drawn from NOAA.
