# Supplementary material for: Attaining freshwater and estuarine-water soil saturation in an ecosystem-scale coastal flooding experiment
Source: Environ Monit Assess. 2023 Feb 24;195(3):425. doi: 10.1007/s10661-022-10807-0 (PMC9958149; doi:10.1007/s10661-022-10807-0)
Supplement: Supplementary file 1 — Supplementary file1 (DOCX 3333 KB) [file 10661_2022_10807_MOESM1_ESM.docx]

**Supplemental Figures:**


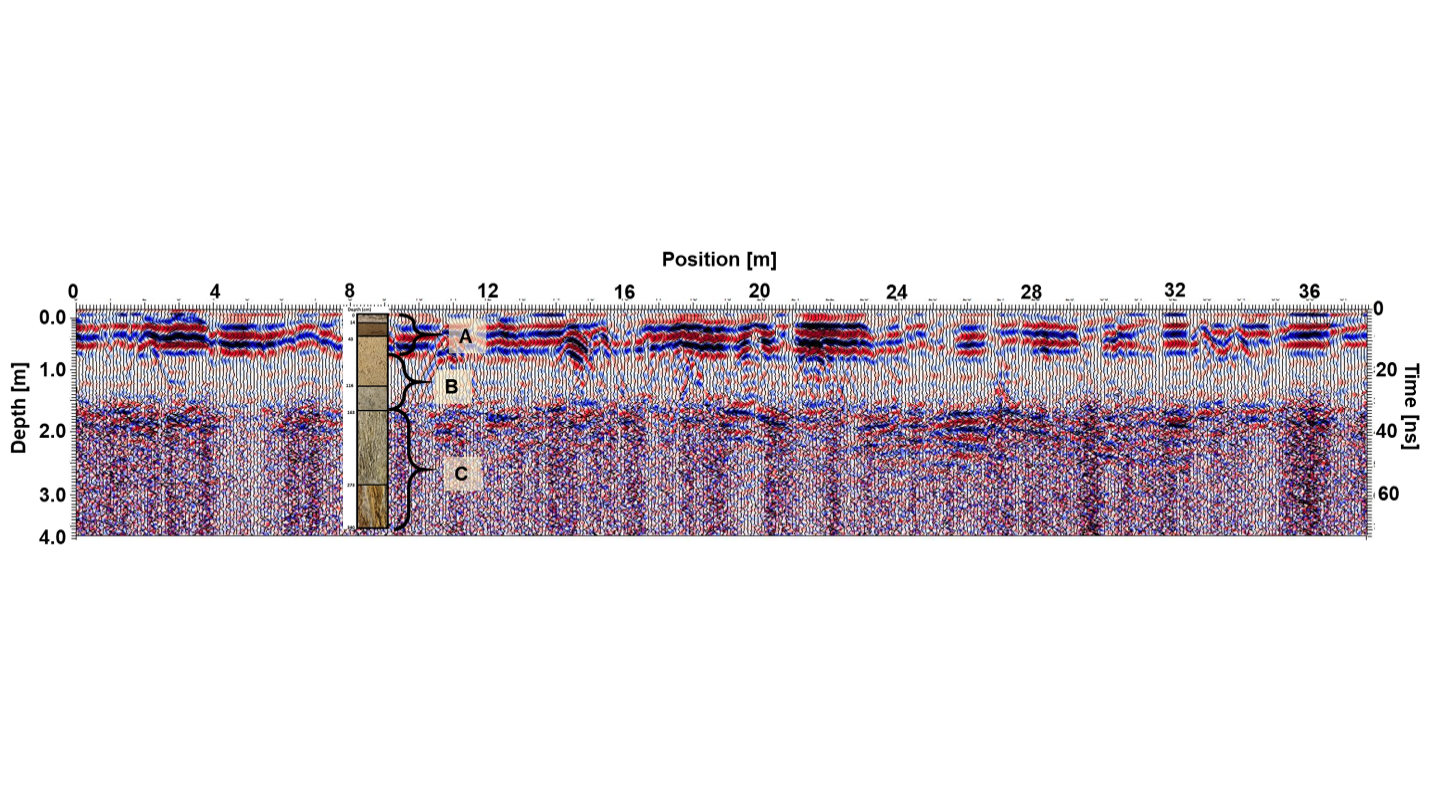


**Supplemental Figure 1.** Rooting zone soils (**A**) consist of sandy loam with high organic matter content and are underlain by silty clay to a depth of 1.6 m (**B**), with clay content increasing with depth. The second unit is underlain by silty sand down to 3.5 m (**C**), with sand content increasing with depth. Soil characterization was performed of an undisturbed 3.5 m deep soil core that was collected along the eastern edge of the Freshwater plot. A ground-penetrating radar transect was co-located along the eastern edge of the Freshwater plot and showed continuity of the sediment units from the soil core.

**
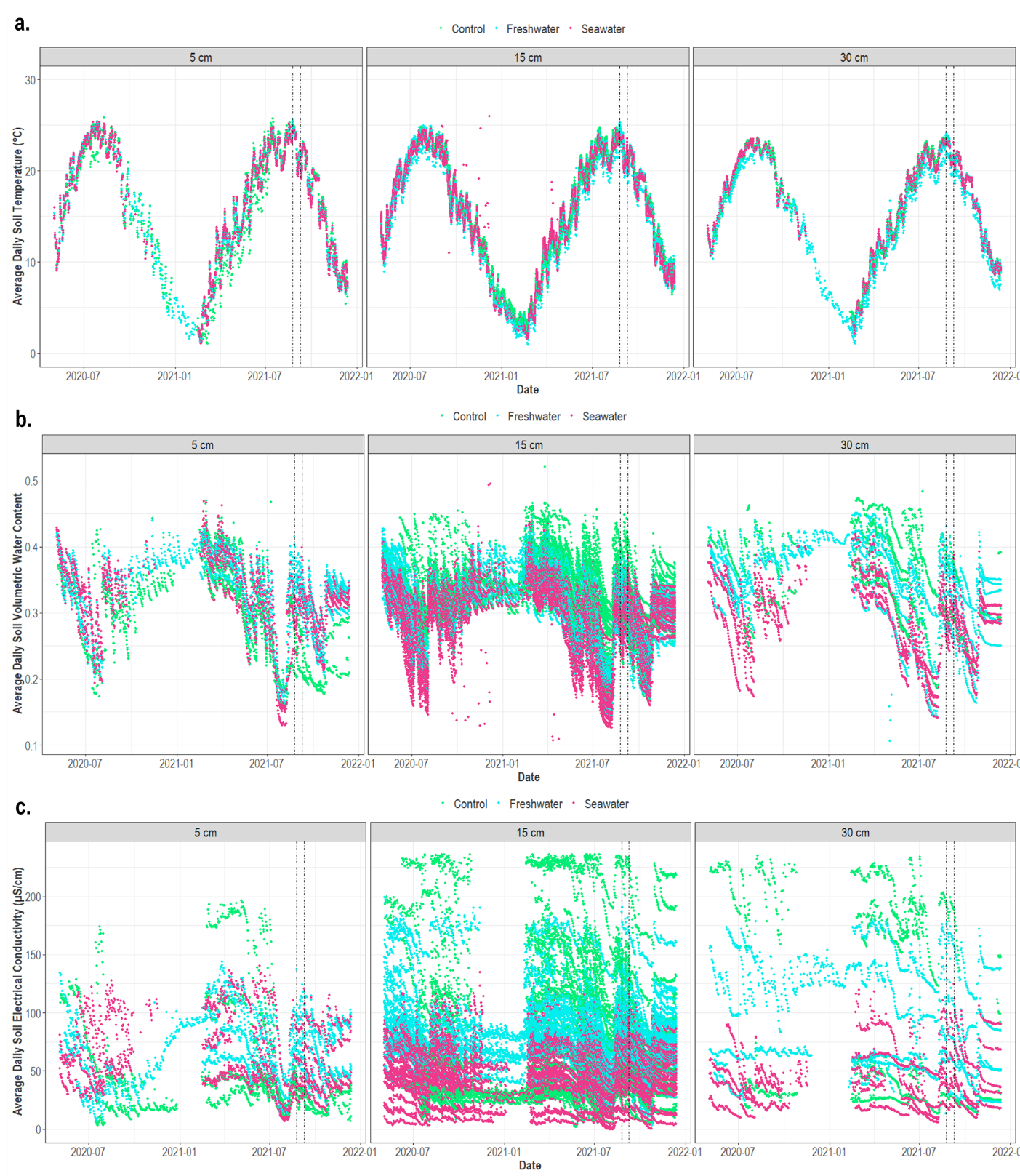
**

**Supplemental Figure 2.** Interannual variation in soil temperature (**a**), volumetric water content (**b**), and electrical conductivity (**c**) at three depths. Simulation events are designated with dotted lines.


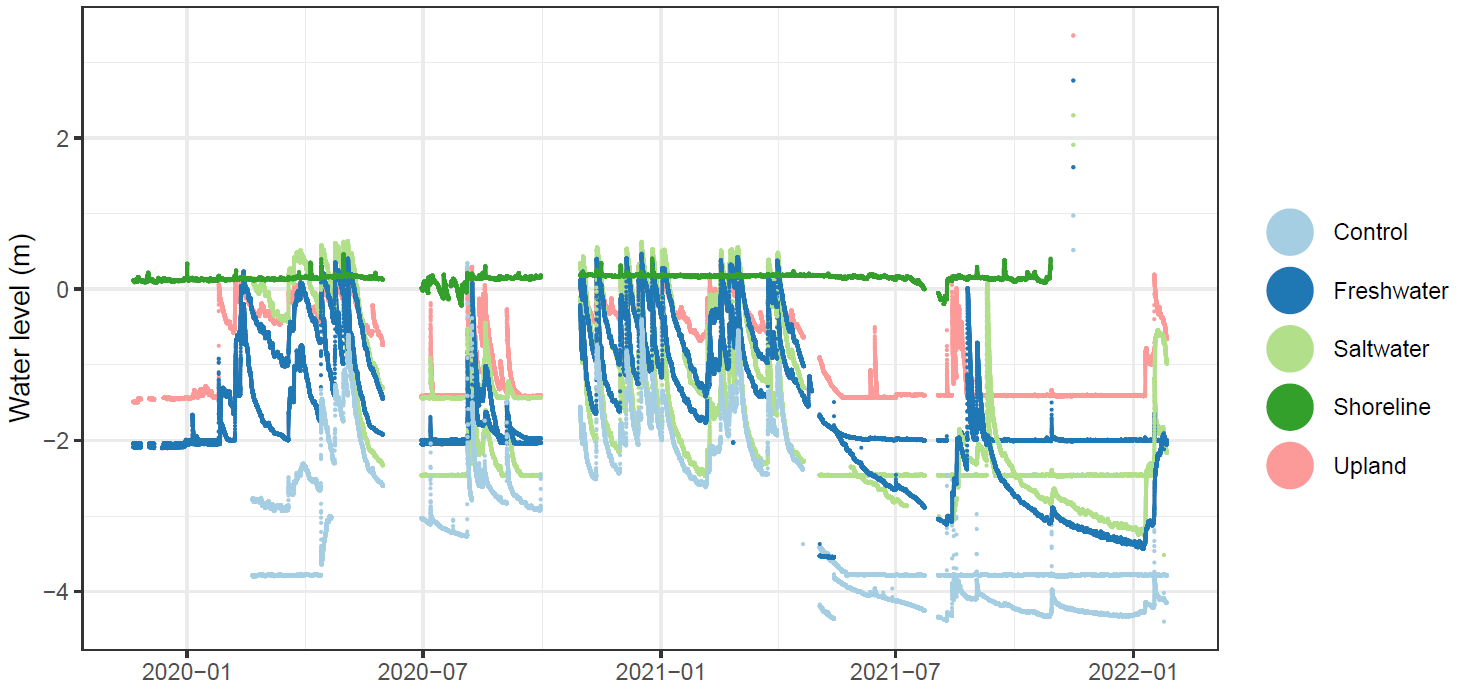


**Supplemental Figure 3.** Interannual variation in groundwater level.


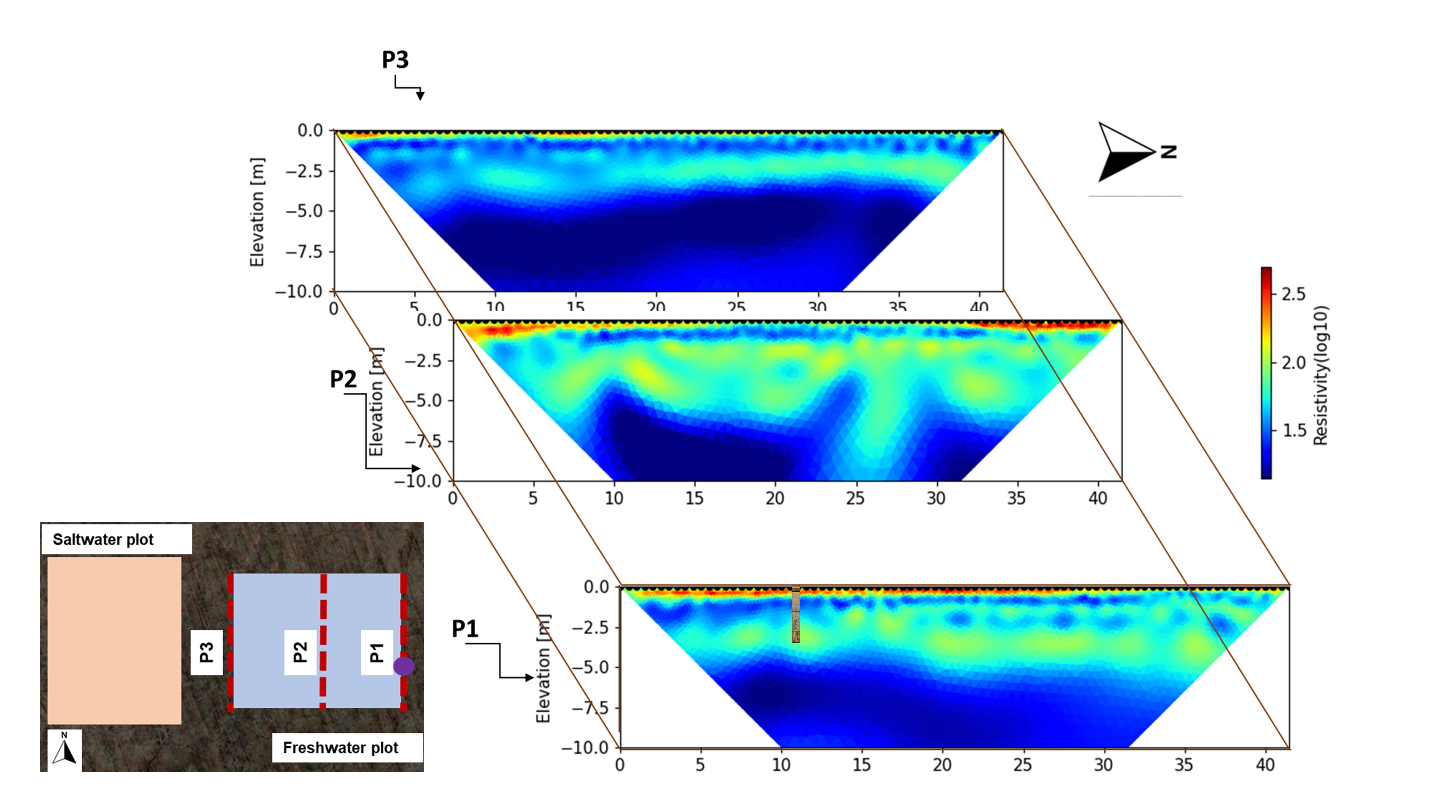


**Supplemental Figure 4.** Electrical resistivity distribution at the Freshwater plot.

**Supplemental Tables:**

**Supplemental Table 1.** Characterization of forest structure within each TEMPEST experimental unit. DBH = diameter at breast height.

| **Plot** | **Number of Trees (DBH ≥ 1 cm)** | **DBH Range (cm)** | **Mean DBH**  **(ave ± st dev)** | **Number of Large Trees**  **(DBH ≥ 20 cm)** |
| --- | --- | --- | --- | --- |
| Control | 140 | 2.0 - 71.0 | 22.3 ± 14.6 | 60 |
| Freshwater | 99 | 1.8 - 79.8 | 24.8 ± 19.7 | 48 |
| Estuarine Water | 159 | 1.4 - 68.4 | 21.5 ± 16.7 | 72 |

**Supplemental Table 2.** Ambient environmental conditions. FW = Freshwater; EW = Estuarine water; NA = Not Applicable.

| **Variable** | **Plot** | **Soil Depth (cm)** | **08/24/2021** | **08/31/2021** | **09/08/2021** |
| --- | --- | --- | --- | --- | --- |
|  |  |  | **24 hours prior to FW plot test** | **24 hours prior to Hurricane Ida** | **24 hours prior to EW plot test** |
|  |  |  |  |  |  |
| **Mean Daily Soil Temperature ± SE (°C)** | Average across all plots | 5 | 24.3 ± 0.20 | 23.8 ± 0.22 | 21.6 ± 0.22 |
|  |  | 15 | 23.8 ± 0.03 | 23.7 ± 0.03 | 21.5 ± 0.03 |
|  |  | 30 | 22.8 ± 0.14 | 23.0 ± 0.13 | 21.0 ± 0.12 |
|  |  |  |  |  |  |
| **Mean Daily Soil VWC ± SE (m^3^/m^3^)** | Average across all plots | 5 | 0.34 ± 0.04 | 0.34 ± 0.04 | 0.34 ± 0.04 |
|  |  | 15 | 0.34 ± 0.01 | 0.34 ± 0.01 | 0.33 ± 0.01 |
|  |  | 30 | 0.36 ± 0.04 | 0.37 ± 0.04 | 0.35 ± 0.04 |
|  |  |  |  |  |  |
| **Mean Daily Water Table Depth ± SE (m)** | Control | NA | 4.07 ± 0.01 | 4.10 ± 0.00 | 4.07 ± 0.00 |
|  | Freshwater | NA | 2.33 ± 0.02 | 1.79 ± 0.04 | 1.98 ± 0.02 |
|  | Estuarine water | NA | 2.17 ± 0.01 | 2.30 ± 0.01 | 2.25 ± 0.01 |

**Supplemental Table 3.** Time to soil saturation and duration by depth for Freshwater and Estuarine-water plot tests. Bold = saturated conditions.

|  | **5 cm** | | **15 cm** | | **30 cm** | |
| --- | --- | --- | --- | --- | --- | --- |
| **Hours post-simulation initiation** | **Mean Hourly FW Plot Soil VWC ± SE (m^3^/m^3^)** | **Mean Hourly EW Plot Soil VWC ± SE (m^3^/m^3^)** | **Mean Hourly FW Plot Soil VWC ± SE (m^3^/m^3^)** | **Mean Hourly EW Plot Soil VWC ± SE (m^3^/m^3^)** | **Mean Hourly FW Plot Soil VWC ± SE (m^3^/m^3^)** | **Mean Hourly EW Plot Soil VWC ± SE (m^3^/m^3^)** |
| 1 | 0.34 ± 0.01 | 0.31 ± 0.01 | 0.32 ± 0.002 | 0.31 ± 0.003 | 0.35 ± 0.01 | 0.29 ± 0.003 |
| 2 | 0.36 ± 0.2 | 0.33 ± 0.01 | 0.35 ± 0.003 | 0.33 ± 0.003 | 0.38 ± 0.01 | 0.35 ± 0.01 |
| 3 | 0.37 ± 0.2 | 0.36 ± 0.01 | 0.36 ± 0.003 | 0.35 ± 0.003 | 0.39 ± 0.001 | 0.36 ± 0.01 |
| 4 | 0.38 ± 0.02 | 0.38 ± 0.02 | 0.37 ± 0.002 | 0.36 ± 0.002 | **0.41 ± 0.01** | 0.36 ± 0.01 |
| 5 | 0.38 ± 0.02 | 0.39 ± 0.02 | 0.38 ± 0.002 | 0.36 ± 0.002 | **0.41 ± 0.01** | **0.37 ± 0.01** |
| 6 | 0.4 ± 0.01 | 0.41 ± 0.01 | **0.39 ± 0.002** | 0.36 ± 0.001 | **0.41 ± 0.01** | **0.37 ± 0.01** |
| 7 | **0.42 ± 0.01** | **0.43 ± 0.01** | **0.39 ± 0.002** | **0.37 ± 0.002** | **0.41 ± 0.01** | **0.37 ± 0.01** |
| 8 | **0.42 ± 0.004** | **0.43 ± 0.01** | **0.39 ± 0.002** | **0.37 ± 0.002** | **0.41 ± 0.01** | **0.37 ± 0.01** |
| 9 | **0.42 ± 0.004** | **0.43 ± 0.01** | **0.39 ± 0.002** | **0.37 ± 0.002** | **0.41 ± 0.01** | **0.37 ± 0.01** |
| 10 | **0.42 ± 0.004** | **0.43 ± 0.01** | **0.39 ± 0.002** | **0.37 ± 0.002** | **0.41 ± 0.01** | **0.37 ± 0.01** |
| 11 | **0.42 ± 0.004** | **0.43 ± 0.01** | **0.39 ± 0.002** | **0.37 ± 0.002** | **0.41 ± 0.01** | **0.37 ± 0.01** |
| 12 | 0.41 ± 0.01 | 0.42 ± 0.01 | **0.39 ± 0.002** | **0.37 ± 0.002** | **0.41 ± 0.01** | **0.37 ± 0.01** |
| 13 | 0.4 ± 0.01 | 0.41 ± 0.01 | 0.38 ± 0.002 | 0.36 ± 0.002 | **0.41 ± 0.01** | **0.37 ± 0.01** |
| 14 | 0.39 ± 0.01 | 0.40 ± 0.01 | 0.37 ± 0.002 | 0.36 ± 0.002 | **0.41 ± 0.01** | **0.37 ± 0.01** |
| 15 | 0.39 ± 0.01 | 0.39 ± 0.01 | 0.37 ± 0.002 | 0.36 ± 0.002 | **0.41 ± 0.01** | **0.37 ± 0.01** |
| 16 | 0.39 ± 0.01 | 0.38 ± 0.01 | 0.37 ± 0.002 | 0.36 ± 0.002 | **0.41 ± 0.01** | **0.37 ± 0.01** |
| 17 | 0.38 ± 0.01 | 0.38 ± 0.01 | 0.36 ± 0.003 | 0.36 ± 0.002 | **0.41 ± 0.01** | **0.37 ± 0.01** |
| 18 | 0.38 ± 0.01 | 0.37 ± 0.01 | 0.36 ± 0.003 | 0.35 ± 0.002 | **0.41 ± 0.01** | **0.37 ± 0.01** |
| 19 | 0.38 ± 0.01 | 0.37 ± 0.01 | 0.36 ± 0.003 | 0.35 ± 0.002 | **0.41 ± 0.01** | 0.36 ± 0.01 |
| 20 | 0.38 ± 0.01 | 0.37 ± 0.01 | 0.36± 0.003 | 0.35 ± 0.002 | **0.41 ± 0.01** | 0.36 ± 0.01 |
| 21 | 0.37 ± 0.01 | 0.36 ± 0.01 | 0.36 ± 0.003 | 0.35 ± 0.002 | **0.41 ± 0.01** | 0.36 ± 0.01 |
| 22 | 0.37 ± 0.01 | 0.36 ± 0.01 | 0.36 ± 0.003 | 0.35 ± 0.002 | **0.41 ± 0.01** | 0.36 ± 0.01 |
| 23 | 0.37 ± 0.01 | 0.36 ± 0.01 | 0.35 ± 0.003 | 0.34 ± 0.002 | **0.41 ± 0.01** | 0.36 ± 0.01 |
| 24 | 0.37 ± 0.01 | 0.36 ± 0.01 | 0.35 ± 0.003 | 0.34 ± 0.002 | 0.39 ± 0.01 | 0.36 ± 0.01 |
